# But How Will We Measure It? Co-Creating Assessments of Outcomes in Age-Friendly Care, PA

**DOI:** 10.1093/geroni/igab046.1224

**Published:** 2021-12-17

**Authors:** Diane Berish

**Affiliations:** Pennsylvania State University, University Park, Pennsylvania, United States

## Abstract

Moving from concept to quantitative measurement can be complex. There were several challenges in co-designing measures to assess the impact of Age-Friendly Care, PA, a geriatric workforce enhancement program. First as a FQHC, our clinical partner had not captured the metrics of interest. Second, the co-developed operational definitions for our metrics should be feasible, relevant, and useful for all project members. Third, funder reporting requirements must also be addressed. Working within this context, we co-created 11 outcome indicators structured around the 4Ms (IHI) now with 9 months of data. EMR changes to make data reportable included measuring opioid misuse mitigation, high-risk medication elimination, cognitive assessment and dementia care management, advanced care planning, care partner presence, annual wellness visit completion, pneumonia vaccination rates, colorectal screening rates, mobility goal tracking, and presence of a caregiver. Work continues around formulating themes to create a reportable mechanism for assessing What Matters.

